# RETRACTION: Inhibitory Effect of Matrine on Blood-Brain Barrier Disruption for the Treatment of Experimental Autoimmune Encephalomyelitis

**DOI:** 10.1155/mi/9829464

**Published:** 2025-05-29

**Authors:** Mediators of Inflammation

RETRACTION: S. Zhang, Q.-C. Kang, Y. Xu, G.-X. Zhang, and L. Zhu, “Inhibitory Effect of Matrine on Blood-Brain Barrier Disruption for the Treatment of Experimental Autoimmune Encephalomyelitis,” *Mediators of Inflammation* 2013, no. 1 (2013): 1–10, https://doi.org/10.1155/2013/736085.

The above article, published online on 08 September 2013 in Wiley Online Library (wileyonlinelibrary.com), has been retracted following the agreement of the journal's Chief Editor, Anshu Agrawal; and John Wiley & Sons Ltd.

The retraction has been agreed following an investigation of the concerns raised by Elizabeth Bik on PubPeer [1], which identified concerns regarding Figures 2a and 6a.

Specifically, similarity has been identified between the features of several panels of Figure 2a with Figure 1c of [2]. In addition, multiple similarities were identified between the features of different panels in Figure 6a.

As a result of the investigation, the data and conclusions of this article are considered unreliable.

The authors had been informed of this retraction but did not provide a response.

## References

[1] Elizabeth M Bik Inhibitory Effect of Matrine on Blood-Brain Barrier Disruption for the Treatment of Experimental Autoimmune Encephalomyelitis. *PubPeer*.

[2] Kan Q.-C., Zhang S., Xu Y.-M., Zhang G.-X., Zhu L. (2014). RETRACTED: Matrine Regulates Glutamate-Related Excitotoxic Factors in Experimental Autoimmune Encephalomyelitis. *Neuroscience Letters*.

